# Evaluation of Glymphatic System Using Diffusion MR Technique in T2DM Cases

**DOI:** 10.3389/fnhum.2020.00300

**Published:** 2020-08-13

**Authors:** Guangwei Yang, Nan Deng, Yi Liu, Yingjiang Gu, Xiang Yao

**Affiliations:** ^1^Hospital (T.C.M) Affiliated to Southwest Medical University, Luzhou, China; ^2^Luzhou People’s Hospital, Luzhou, China; ^3^Department of Radiology, Xiang’an Hospital of Xiamen University, Xiamen, China

**Keywords:** glymphatic system, type 2 diabetes mellitus, MRI, diffusion tensor, perivascular space

## Abstract

**Objective**: We aimed to evaluate the activity of the human glymphatic system in type 2 diabetes mellitus (T2DM) using diffusion tensor image analysis along with the perivascular space (DTI-ALPS).

**Methods**: Diffusion tensor images were acquired to calculate the diffusivities in the direction of the x-axis (right-to-left; Dx), y-axis (anterior-to-posterior; Dy), and z-axis (inferior-to-superior; Dz) of the plane of the lateral ventricle body in 20 patients with type 2 diabetes and 10 people in a control group. We evaluated the diffusivity along with the perivascular spaces, as well as the projection fibers and association fibers, separately. The analysis along the perivascular space (ALPS-index) was defined as the mean (Dxpro, Dypro)/mean (Dypro, Dzasc), where the Dxpro and Dxasc were the Dx values in the projection and association fiber areas, respectively.

**Results**: There were significant differences in diffusivity along the projection fibers and the association fibers among the groups. The significant differences among the groups along the perivascular spaces, shown as the ALPS-index and medical history of T2DM, indicating lower water diffusivity along the perivascular space concerning type 2 diabetes severity, was also observed.

**Conclusion**: Lower diffusivity along the perivascular space on DTI-APLS can reflect impairment of the glymphatic system in T2DM. This study showed that the activity of the glymphatic system could be evaluated by diffusion tensor image analysis.

## Introduction

The glymphatic lymphatic system is a waste excretion system recently discovered in the brain, which involves the movement of cerebrospinal fluid (CSF) along the perivascular space. The system promotes the removal of soluble proteins, including amyloid-β (Aβ) and metabolites, as well as the distribution of glucose, lipids, amino acids, and neuromodulators (Iliff et al., 2012, 2013; Jessen et al., 2015). Besides, the system is also closely related to the pathophysiology of various neurological diseases, such as Alzheimer’s disease (AD; Xie et al., 2013).

Type 2 diabetes mellitus (T2DM) is a serious health problem and an established risk factor for cognitive decline in elderly people. The causative roles of the DM-associated cerebrovascular dysfunction and neurodegenerative mechanism following abnormal glycemia and insulinemia have been described in the pathogenesis of cognitive impairment (McCrimmon et al., 2012; Mayeda et al., 2015; Moheet et al., 2015). However, the pathological changes in the central nervous system in patients with diabetes are usually subtle and multifactorial. Therefore, obtaining imaging biomarkers of DM-associated glymphatic impairment and cognitive decline may help identify high-risk patients and evaluate potential treatments.

**Figure 1:**
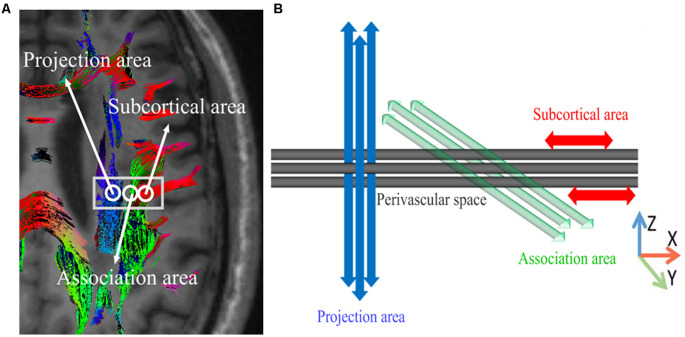
The concept of the diffusion tensor image analysis along with the perivascular space (DTI-ALPS) method for perivascular diffusion. **(A)** The DTI superimposed color display shows the distribution of projection fibers (z-axis: blue), association fibers (y-axis: green), and subcortical fibers (x-axis: red). Three regions of interest (ROIs) were placed in the area with projection fibers (projection area), association fibers (association area), and subcortical fibers (subcortical area) to measure the diffusivities in three directions (x, y, and z). **(B)** Schematic diagram showing the relationship between the direction of the perivascular space (gray cylinder) and the direction of the fibers. Note that the direction of the perivascular space is perpendicular to the projection and the association fibers. The figure has been reprinted with permission from Taoka et al. (2017).

Previous studies using glymphatic MRI with intrathecal contrast agent administration revealed delayed glymphatic clearance and transependymal migration of the contrast agents in idiopathic normal pressure hydrocephalus. However, this method is invasive and requires multiple MRI acquisitions before and after the intrathecal injection of contrast agents (Iliff et al., 2012). The purpose of this study was to assess the feasibility of a non-invasive method that uses diffusion images to assess the activity of the lymphatic system in the human brain. In this method, we measured the movement of water molecules in the direction of the perivascular space using the diffusion tensor method. At the level of the lateral ventricle body, the medullary veins run perpendicularly to the ventricular wall (Okudera et al., 1999), and the perivascular space runs in the same direction as the medullary vein, which is in the right-left direction (x-axis). On the plane of this area, the projection fibers are mainly in the head-feet direction, close to the lateral ventricle, and the superior longitudinal fascicles (SLFs), representing association fibers in the current study, run in an anterior-posterior direction outside the projection fibers. Outside the SLFs, subcortical fibers run mainly in the right-left direction of the subcortical area. Therefore, in this area, the perivascular space is perpendicular to the projection fibers and SLFs. The confirmation of the perivascular space and the main fibers in this area allow almost independent analysis of the diffusivity in the direction of the perivascular space because the main fiber tracts do not run in a direction parallel to the perivascular space. When there is a histological change in the left-right direction (x-axis), it will also affect both the projection and the association fibers. Therefore, a change observed in both fiber bundles suggests that at least a part of this change came from the pathology involving the perivascular space, namely the glymphatic system. We aimed to investigate the pathological changes of the lymphatic system in patients with T2DM using this new method of diffusion tensor image (DTI) analysis (Figure 1).

## Materials and Methods

### Patients

This study was evaluated and approved by our Institutional Review Board. We studied 30 patients (14 males and 17 females; mean 66 years old; age range 56–75 years ), 10 T2DM patients with a history of more than 10 years, 10 T2DM patients with a history of fewer than 10 years, and 10 normal people in the control group. All subjects were right-handed.

### MRI Protocol

Diffusion imaging was acquired by using a 3.0-T clinical scanner (Magnetom Verio, Siemens AG, Erlangen, Germany). DTI sets with *b* = 0, *b* = 1,000, and *b* = 2,000 s/mm^2^ (echo planer, *TR* = 6,600 ms, *TE* = 89 ms, *MPG* = 30 directions, *FOV* = 230 mm, matrix = 94 × 94, and slice thickness = 5 mm) were acquired simultaneously.

### Analysis Along the Perivascular Space (ALPS) Index

The DTI data were handled by FSL ver. 5.0.9^1^. The software generates images of the diffusion tensor calculations, including a color-coded fractional anisotropy (FA) map and a diffusivity map. First, the DTI raw data were corrected for head motion and eddy current-induced geometrical distortions. We used BET (brain extraction toolbox) to create a brain template for head motion correction. In this experiment, we set up a strict head motion parameter. The images with head movement greater than 0.2 mm and rotation greater than 0.2 mm are excluded. The FDT tool provided by FSL is used for eddy current correction of DTI image. Then the *b* = 0 image of each subject was skull-stripped with the brain extraction tool (BET v2.1).

Also, the diffusivity in the x-axis, y-axis, and z-axis directions on each image can be calculated by the software. This function was used in the diffusion tensor image analysis along with the perivascular space (DTI-ALPS) method analysis, evaluating the diffusivity along the direction of the perivascular space compared with the diffusivity of projection fibers and association fibers on a slice at the level of the lateral ventricle body (Figure 1). At this level, the direction of the perivascular space is perpendicular to the ventricle wall, so it is mainly in the right-left direction (x-axis) on the axial plane. This direction is also perpendicular to the direction of the projection fiber (mainly on the z-axis) and the associated fiber (mainly on the y-axis; Figure 1B). Therefore, in regions with projection/association fibers, the diffusivity along the x-axis will at least partially, represent the diffusivity along the perivascular space. A 5-mm diameter spherical region of interest (ROI) was placed in the projection fibers area (blue in Figure 1A), the association fibers area (green in Figure 1A), and the area of subcortical fibers (red in Figure 1A) in the left hemisphere. For each area, we calculated the diffusivity of three groups along the x-axis, y-axis, and z-axis. We only measured in the left hemisphere because all of the subjects were right-handed.

### ALPS-Index Determination Method

The ALPS-index was calculated to assess the activity of the glymphatic system in individual cases. The index value was derived from the ratio of the two diffusivity value sets that are perpendicular to the main fibers in the tissue: that is, the ratio of the average values of the x-axis diffusivity in the area of the projection fibers (Dxproj) and the x-axis diffusivity in the area of the association fibers (Dxassoc) to the average value of the y-axis diffusivity in the area of the projection fibers (Dyproj) and the z-axis diffusivity (Dzacoc) of the association fibers area, as shown below:

ALPS index = mean (Dxproj, Dxassoc)/mean (Dyproj, Dzassoc).

In the projection fibers area, the main fibers run along the z-axis, and the x-axis and y-axis are perpendicular to the main fibers. Similarly, in the association fibers area, the main fibers run in the direction of the y-axis, and both the x-axis and z-axis are perpendicular to the main fibers. We made the above analyses with *b* = 1,000 and *b* = 2,000 s/mm^2^. The main difference in the behavior of the water molecules between the x-axis diffusivity in both areas (Dxproj and Dxassoc) and the diffusivity perpendicular to them (Dyproj and Dzassoc) is the presence of the perivascular space.

### Statistical Analysis

A paired *t*-test and Wilcoxon rank-sum test were used for evaluating patient characteristics. The statistical significance levels of the report were two-sided, with the statistical significance set at 0.05. All of the data were analyzed with SPSS statistical software (version 17.0, SPSS).

## Results

**Figure 2:**
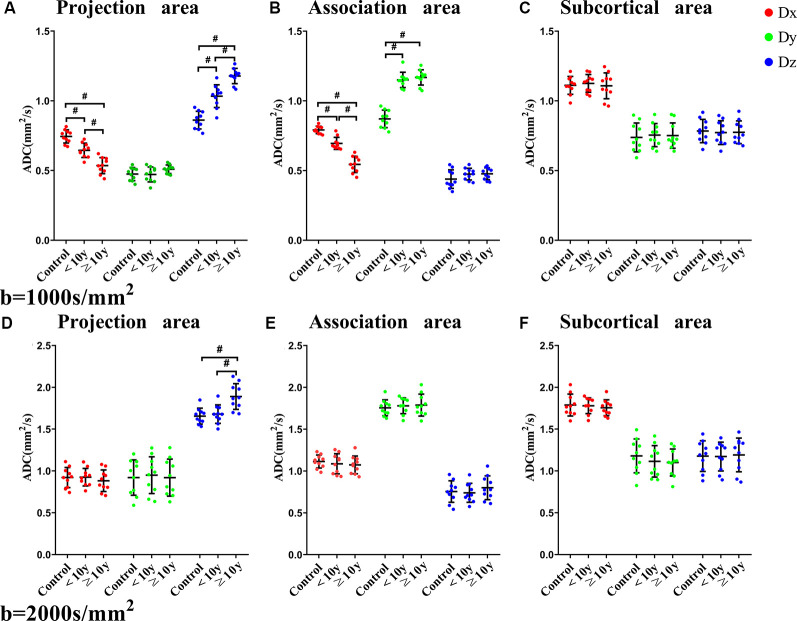
The relationship between directional diffusivity and the history of T2DM. Relationship between a history of T2DM and diffusivities for the three directions of the three areas [projection **(A,D)**, association **(B,E)**, subcortical **(C,F)**] with the *b* = 1,000 s/mm^2^ datasets **(A,B,C)** and the *b* = 2,000 s/mm^2^ datasets **(D,E,F)**. The diffusivity of the x-axis is plotted in red, the y-axis is plotted in green, and the z-axis is plotted in blue. Statistically, significant differences are indicated by #.

The characteristics of the control, <10 years (y) T2DM, and ≥10 y T2DM groups are summarized in Table 1. The relationship between T2DM medical history and diffusivity in the three areas (projection, association, subcortical) for the three directions (x, y, and z) are shown in Figure 2. In the *b* = 1,000 s/mm^2^ datasets in the projection area, we found that there was a significant difference in diffusivity along the perivascular space (x-axis) between <10 y T2DM group and ≥10 y T2DM group (*P* < 0.001), as well as control group with <10 y T2DM group (*P* < 0.001). By contrast, ≥ the 10 y T2DM group was significantly higher than the <10 y T2DM group and control group in diffusivity along the projection fiber (z-axis; *P* < 0.001). There was no significant difference between the three groups in the anterior-posterior direction (y-axis) along the projection fiber. There was a significant difference between <10 y T2DM group and ≥10 y T2DM group in diffusivity along the association area (x-axis; *P* < 0.001), as well as the control group with <10 y T2DM group (*P* < 0.001). At the same time, ≥ the 10 y T2DM group and <10 y T2DM group were significantly higher than the control group in diffusivity along the association fiber (y-axis; *P* < 0.001). There was no significant difference between the three groups in the head-feet direction (z-axis) along the association fiber. Diffusivity in the three groups was no significant difference in the x-axis, y-axis, and z-axis along the subcortical area. In the *b* = 2,000 s/mm^2^ measurement, except for the diffusivity along the projection fiber (z-axis), which showed a significant difference between ≥10 y T2DM group and <10 y T2DM group in the projection area (*P* < 0.001), there was no statistically significant difference between three groups.

**Table 1:** Patient characteristics.

	Control	<10 y	≥10 y	*P*-values		
	(*n* = 10)	(*n* = 10)	(*n* = 10)	Control vs. <10 y	Control vs. ≥10 y	<10 y vs. ≥10 y
Age (years, y)	65.7 ± 8.4	65.7 ± 9.4	65.3 ± 7.3	1	1	1
Sex (Female, Male)	5, 5	6, 4	4, 6	1	1	1
Postprandial glucose (mmol/l)	5.5	6.4	7.6	<0.001	<0.001	<0.001
HbA1c%	4.7	5.8	6.2	<0.001	<0.001	<0.165
Treatment (insulin, oral antidiabetic drugs)	None	4, 6	6, 4	1	1	1

**Figure 3:**
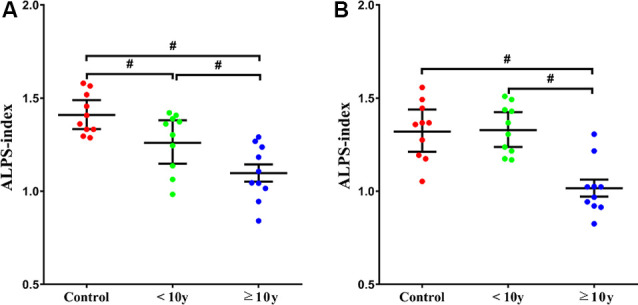
The relationship between the ALPS-index and history of T2DM [**(A)** *b* = 1,000 s/mm^2^, **(B)** *b* = 2,000 s/mm^2^]. ALPS-index = average (Dxproj, Dxassoc)/average (Dyproj, Dzassoc). Statistically, significant differences are indicated by #.

In the *b* = 1,000 s/mm^2^ datasets, we found that there was a significant difference in the ALPS-index values between <10 y T2DM group and ≥10 y T2DM group (*P* < 0.001), as well as control group with <10 y T2DM group (*P* < 0.001; Figure 3A). In the *b* = 2,000 s/mm^2^ measurement, control group and <10 y T2DM group were significantly higher than the ≥10 y T2DM group in the ALPS-index values (*P* < 0.001; Figure 3B).

## Discussion

The incidence of cognitive dysfunction and AD is higher in diabetic patients, suggesting that diabetes plays an important role in the development of cognitive dysfunction and AD. One of the important neuropathological mechanisms of cognitive dysfunction in diabetic patients is the accumulation of disaggregated proteins, including senile plaques composed of Aβ, which has been proved to affect cognitive function (Plog et al., 2015; Baglietto-Vargas et al., 2016). Previous studies have shown that the glymphatic system regulates the clearance of Aβ, and provided relevant evidence to link the impairment of interstitial fluid (ISF) clearance caused by DM with cognitive impairment (Mehla et al., 2014; Vandal et al., 2014). The paravascular space likely activates a series of inflammatory reactions, leading to neurovascular disruption, including an increase in the paravascular space, pathological characteristics of the diabetic brain, and other dementia diseases (Doubal et al., 2010; Wardlaw et al., 2013). Therefore, the accumulation of Aβ deposits and neurovascular disruption after glymphatic impairment may promote a positive feedback loop, further adversely affecting the glymphatic system function in the diabetic brain.

The glymphatic system has been observed through tracing studies in animal experiments. Iliff et al. (2012) found that CSF entered the brain rapidly along the cortical pial arteries after labeling the CSF by injecting a fluorescent tracer into the cistern Magna CSF. The dynamic characteristics of the glymphatic system were observed for the first time in mice by two-photon microscopy. In addition to fluorescent materials, intrathecal injections of gadolinium-based contrast agents (GBCAs) were used as tracers for later animal studies (Tali et al., 2002; Öner et al., 2017). However, except for several well-designed studies and an accident report, no studies of the intrathecal administration of tracers in humans have been conducted. Injection of GBCAs into the CSF can cause high T1 intensity in the globus pallidus and dentate nucleus, which strongly suggests that the glymphatic system is involved in gadolinium deposition in the globus pallidus and dentate nucleus (Kartamihardja et al., 2016; Robert et al., 2016). However, no method has been established to track intravenous injections of GBCAs with MRI. Also, even if a tracking method is established to evaluate the human lymphatic system in living humans, it will take hours to track the distribution of a tracer in the brain, and it is impossible to monitor the activity of the lymphatic system in real-time (Tali et al., 2002). Therefore, in addition to tracer studies, other monitoring methods are needed to assess the glymphatic system.

The DTI-ALPS method was used in this study to assess diffusion images, which can be obtained in a few minutes, and it was possible to monitor the status of the glymphatic system over time. The diffusivity along the direction of the perivascular space, compared with the direction of projection fibers and association fibers on a slice at the level of the lateral ventricle body, were evaluated by the DTI-ALPS method. Medullary arteries and veins are the blood vessels of the brain parenchyma, accompanied by the space around the perivascular space, which is the main drainage path of the glymphatic system. We found that outside the projection fibers at the level of the lateral ventricle body, the perivascular space runs in a right-left direction (Dx-axis), projection fibers run in a head-foot (Dz-axis) direction, and association fibers run in an anterior-posterior (Dy-axis) direction.

Our results showed that the diffusivity along the projection fibers and association fibers of ≥ the 10 y T2DM group was lower than <10 y T2DM group and control group. This result is consistent with the white matter degeneration of projection or association fibers caused by T2DM shown in a previous report, indicating that in the areas where projection or association fibers are dominant, water diffusivity in the direction of the perivascular space is damaged, which is related to the severity of T2DM (Huang et al., 2012). T2DM is a common feature of patients with cortical atherothrombotic embolism and lacunar stroke (Jackson et al., 2010; Giwa et al., 2012). It changes the structure of the arterioles and increases the perivascular space. Microvascular and macrovascular damage can be induced by T2DM, increasing the risk of developing small vessel disease with enlargement of the perivascular space (Morton and Schwartz, 2011; Prasad et al., 2014; Eide and Ringstad, 2015). Inflammation may also play an important role in the expansion of the perivascular space (Rouhl et al., 2012). Previous studies have shown that active inflammation in multiple sclerosis and lacunar stroke increased the perivascular space (Wuerfel et al., 2008; Bailey et al., 2012). Infiltration of inflammatory cells was found in the penetrating arterioles and perivascular tissues of patients with small vessel disease. The increase in plasma inflammatory markers in patients with lacunar stroke was related to the high intensity of the white matter and the progress of lacunar infarction (Bailey et al., 2012). The expansion of the paravascular space could also be a factor reducing the clearance of GBCAs caused by the stagnation of glymphatic transport. Our results are consistent with the impaired activity of the glymphatic system in T2DM suggested by experiments in rats that are interpreted as decreased tissue density in these tracts in T2DM cases (Huang et al., 2012). We conducted evaluations with two different *b* values, where *b* = 1,000 s/mm^2^ was the standard *b* value, and *b* = 2,000 s/mm^2^ was the higher *b* value. To ensure a better signal to noise ratio, we did not choose a very high *b* value, such as *b* = 3,000 s/mm^2^. When the value of b is low, the influence of water molecules with higher motivity becomes dominant; higher diffusivity or motivity of the water molecules in the perivascular space could have a greater influence with a lower *b* value. Another possible reason for the difference between the *b* = 1,000 and *b* = 2,000 s/mm^2^ results is the higher signal to noise ratio obtained in the *b* = 1,000 s/mm^2^ measurement.

We calculated the ALPS-index to assess the lymphatic system activity in individual cases. In this index, we assumed that the ratio of the x-axis diffusivity of the projection fiber and association fibers area (Dxproj and Dxassoc) to the diffusivity perpendicular to them (Dyproj and Dzassoc) would represent the effect of water diffusion along the perivascular space, reflecting the activity of the glymphatic system in the individual cases. When the ratio is close to 1, it means that the influence of the water diffusion along the perivascular space is minimal. The higher the ratio, the more water diffusivity along the perivascular space. In our results, ALPS-index in the *b* = 1,000 s/mm^2^ measurement of ≥10 y T2DM group was lower than <10 y T2DM group and control group. The results show that the ALPS-index could be used to evaluate the glymphatic system activity in individual cases. The results also indicated that all of the patients with a longer T2DM history had lower ALPS-indexes. This may indicate that the study patients with severe T2DM had a damaged glymphatic system, almost without exception.

Our study had several limitations. Currently, our method can only calculate diffusivity in the Dx, Dy, and Dz axes. Therefore, the area outside the lateral ventricle along the lateral ventricle body plane is the only place where the diffusivity along the direction of the perivascular space can be independently assessed. In areas where the perivascular space does not run in Dx, Dy, or Dz axes directions or areas in which the perivascular space and the direction of the predominant fiber tract are parallel, it is impossible to perform an isolated evaluation of the diffusivity along the perivascular space. Another limitation of our study is that the ROI was placed manually, which may have introduced a subjective factor into our measurements. We tried to place the ROI as objectively as possible. The small number of subjects and the fact that the study was conducted in a single institution are other limitations. A further study with a larger study group is needed to evaluate glymphatic system dysfunction by other factors. We will apply our method to other neurological disorders, such as gliomas and Alzheimer’s disease. Also, we did not consider any other physiological factors, including cerebral perfusion or pulsatile motion. However, we performed diffusion imaging with *b* = 1,000 and *b* = 2,000 s/mm^2^ because this high motion gradient is expected to cancel out the rather macroscopic physiological status.

In conclusion, our results using the DTI-ALPS method showed that impaired water diffusivity was associated with the severity of T2DM. Therefore, lower diffusivity along the perivascular space or lower ALPS-index seems to reflect the damage of the glymphatic system. The DTI-APLS method can be used to assess the activity of the glymphatic system, and the ALPS-index can be used to assess conditions that affect the activity of the glymphatic system in individual cases.

## Data Availability Statement

All datasets presented in this study are included in the article.

## Ethics Statement

The studies involving human participants were reviewed and approved by Xiang’an Hospital of Xiamen University. The patients/participants provided their written informed consent to participate in this study. Written informed consent was obtained from the individual(s) for the publication of any potentially identifiable images or data included in this article.

## Author Contributions

All authors listed have made a substantial, direct and intellectual contribution to the work, and approved it for publication.

## Conflict of Interest

The authors declare that the research was conducted in the absence of any commercial or financial relationships that could be construed as a potential conflict of interest.

## Footnotes

^1^https://fsl.fmrib.ox.ac.uk/
