# Corrigendum: Evaluation of Glymphatic System Using Diffusion MR Technique in T2DM Cases

**DOI:** 10.3389/fnhum.2020.616400

**Published:** 2021-01-26

**Authors:** Guangwei Yang, Nan Deng, Yi Liu, Yingjiang Gu, Xiang Yao

**Affiliations:** ^1^Hospital (T.C.M) Affiliated to Southwest Medical University, Luzhou, China; ^2^Luzhou People's Hospital, Luzhou, China; ^3^Department of Radiology, Xiang'an Hospital of Xiamen University, Xiamen, China

**Keywords:** glymphatic system, type 2 diabetes mellitus, MRI, diffusion tensor, perivascular space

In the original article, there was a mistake in the legend for Figure 1 as published. The figure, which was previously published in the article cited below from the Japanese Journal of Radiology, was reused without appropriate acknowledgment in our article. The correct legend appears below.

Figure 1 The concept of the diffusion tensor image analysis along with the perivascular space (DTI-ALPS) method for perivascular diffusion. (A) The DTI superimposed color display shows the distribution of projection fibers (z-axis: blue), association fibers (y-axis: green), and subcortical fibers (x-axis: red). Three regions of interest (ROIs) were placed in the area with projection fibers (projection area), association fibers (association area), and subcortical fibers (subcortical area) to measure the diffusivities in three directions (x, y, and z). (B) Schematic diagram showing the relationship between the direction of the perivascular space (gray cylinder) and the direction of the fibers. Note that the direction of the perivascular space is perpendicular to the projection and the association fibers. The figure has been reprinted with permission from Taoka et al. (2017).

The authors apologize for this error and state that this does not change the scientific conclusions of the article in any way. The original article has been updated.
